# (*Z*)-2-[Meth­oxy(phen­yl)methyl­idene]-3,4,5-trimethyl-2,3-dihydro-1,3-thia­zole

**DOI:** 10.1107/S1600536812034137

**Published:** 2012-08-04

**Authors:** Biplab Maji, Herbert Mayr, Peter Mayer

**Affiliations:** a Ludwig-Maximilians-Universität, Department, Butenandtstrasse 5–13, 81377 München, Germany

## Abstract

In the title compound, C_14_H_17_NOS, the plane defined by the bridging methyl­ene C atom and its three substituents makes dihedral angles of 14.37 (8)° with the heterocycle and 26.17 (8)° with the phenyl ring, while the dihedral angle between the heterocycle and the phenyl ring is 36.29 (7)°. In the crystal, mol­ecules are linked by C—H⋯π contacts.

## Related literature

For chemical background, see: Ukai *et al.* (1943 ▶); Enders *et al.* (2007 ▶); Biju *et al.* (2011 ▶); Breslow (1958 ▶). For a related structure, see: Reisser *et al.* (2003 ▶).
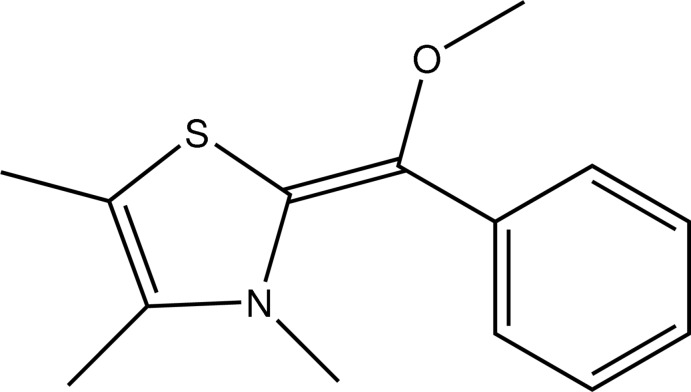


## Experimental

### 

#### Crystal data

C_14_H_17_NOS
*M* *_r_* = 247.36
Monoclinic, 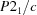

*a* = 15.9660 (7) Å
*b* = 6.8902 (3) Å
*c* = 12.1520 (6) Å
β = 103.381 (5)°
*V* = 1300.54 (10) Å^3^
*Z* = 4
Mo *K*α radiation
μ = 0.23 mm^−1^
*T* = 173 K
0.35 × 0.25 × 0.17 mm

#### Data collection

Oxford Diffraction Xcalibur diffractometer
Absorption correction: multi-scan (*CrysAlis PRO*; Oxford Diffraction, 2009 ▶) *T*_min_ = 0.953, *T*_max_ = 1.000
9041 measured reflections
2637 independent reflections
2023 reflections with *I* > 2σ(*I*)
*R*_int_ = 0.029

#### Refinement

*R*[*F* ^2^ > 2σ(*F* ^2^)] = 0.036
*wR*(*F* ^2^) = 0.104
*S* = 1.08
2637 reflections
158 parameters
H-atom parameters constrained
Δρ_max_ = 0.29 e Å^−3^
Δρ_min_ = −0.19 e Å^−3^

### 

Data collection: *CrysAlis PRO* (Oxford Diffraction, 2009 ▶); cell refinement: *CrysAlis PRO*; data reduction: *CrysAlis PRO*; program(s) used to solve structure: *SIR99* (Altomare *et al.*, 1999 ▶); program(s) used to refine structure: *SHELXL97* (Sheldrick, 2008 ▶); molecular graphics: *ORTEP-3* (Farrugia, 1997 ▶) and *PLATON* (Spek, 2009 ▶); software used to prepare material for publication: *SHELXL97*.

## Supplementary Material

Crystal structure: contains datablock(s) I, global. DOI: 10.1107/S1600536812034137/su2484sup1.cif

Structure factors: contains datablock(s) I. DOI: 10.1107/S1600536812034137/su2484Isup2.hkl

Supplementary material file. DOI: 10.1107/S1600536812034137/su2484Isup3.cml

Additional supplementary materials:  crystallographic information; 3D view; checkCIF report

## Figures and Tables

**Table 1:** Hydrogen-bond geometry (Å, °) *Cg*1 is the centroid of the C5–C10 ring.

*D*—H⋯*A*	*D*—H	H⋯*A*	*D*⋯*A*	*D*—H⋯*A*
C8—H8⋯*Cg*1^i^	0.95	2.83	3.6324 (16)	142
C13—H13*B*⋯*Cg*1^ii^	0.98	2.74	3.5657 (15)	143

Symmetry codes: (i) 
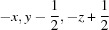
; (ii) 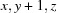.

**Fig. 1.**
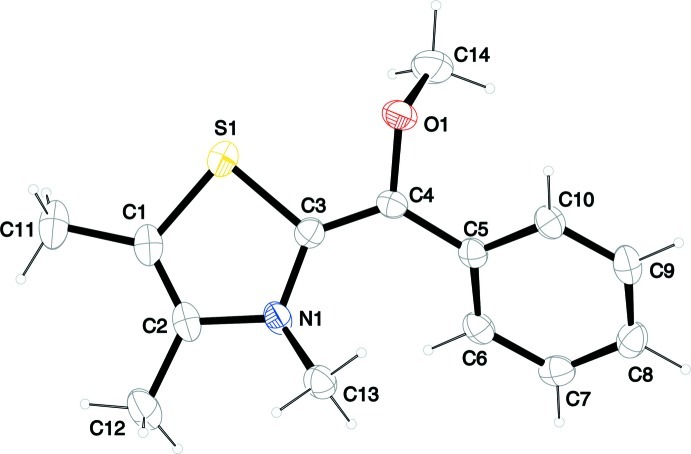
The molecular structure of the title molecule, with atom labelling. Displacement ellipsoids are drawn at 50% probability level.

**Fig. 2.**
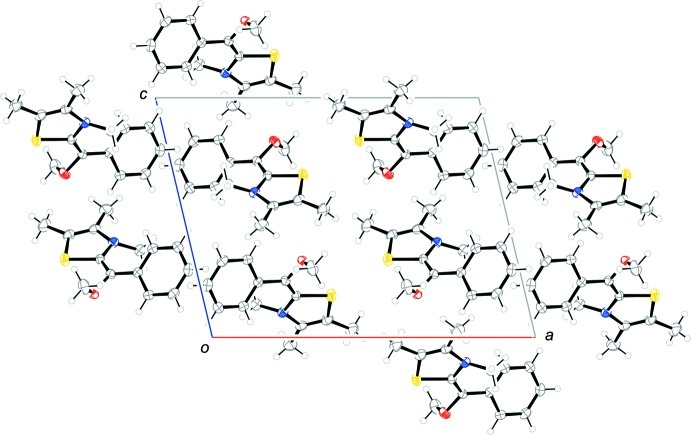
The crystal packing of the title compound viewed along the b axis.

## supplementary crystallographic information

### Comment

Thiazolium ions (Ukai *et al.*, 1943) are known to catalyze benzoin condensations of aldehydes (Enders *et al.*, 2007; Biju *et al.*, 2011) in presence of a base. An acyl anion equivalent, the so-called Breslow intermediate (Breslow, 1958) was proposed to be the key intermediate of these transformations. To understand the structure of these intermediates we now report the X-ray analysis of the *O*-methyl-protected Breslow intermediate derived from 3,4,5-trimethylthiazolium ion and benzaldehyde.

The molecular structure of the title compound is shown in Fig. 1. The exocyclic double bond has a length of 1.349 (2) Å which is comparable to that observed for a related structure [1.353 Å; Reisser *et al.*, 2003]. The endocyclic double bond length is 1.330 (2) Å [1.332 Å; Reisser *et al.*, 2003]. The angle sum around the methylene carbon atom, C4, amounts to 360° resulting in a trigonal planar environment of the methylene atom. However, this mean plane (C4/O1/C3/C5) is not coplanar with either the plane of the heterocycle (S1/N1/C1-C3) or the plane of the phenyl ring (C5-C10). The corresponding dihedral angles are 14.37 (8)° and 26.17 (8)°, respectively. The dihedral angle between the heterocycle and the phenyl ring is 36.29 (7)°.

In the crystal, molecules are linked via C–H···π contacts (Table 1 and Fig. 2).

### Experimental

A solution of 2-(methoxy(phenyl)methyl)-3,4,5-trimethylthiazolium trifluoromethanesulfonate (397 mg, 1.00 mmol) in THF (6 ml) was added dropwise to a stirred suspension of NaH (36 mg, 1.5 mmol) in dry THF (5 ml) at -20 °C under nitrogen, and the reaction mixture was allowed to stir for 36 h in the dark. After warming to room temperature, the solvent was removed under vacuum, and the residue was suspended in dry toluene (20 ml) and filtered through a celite pad under nitrogen. Then the solvent was evaporated to give 205 mg (0.829 mmol, 83%) of the title compound as 2:1 mixture of *Z*:*E* isomers. Crystals of the title compound suitable for X-ray diffraction analysis were grown by slow evaporation of a solution in *n*-pentane under nitrogen.

### Refinement

The C-bound H atoms were included in calculated positions and treated as riding atoms: C-H = 0.95 and 0.98 Å for CH and CH_3_ -atoms, respectively, with U_iso_(H) = k × U_eq_(C), where k = 1.5 for CH_3_ H atoms and = 1.2 for other H atoms. The methyl groups were allowed to rotate along the C–*X* bonds (*X* = C, O, N) to best fit the experimental electron density.

### Crystal data

**Table:**

C_14_H_17_NOS	*F*(000) = 528
*M**_r_* = 247.36	*D*_x_ = 1.263 (1) Mg m^−^^3^
Monoclinic, *P*2_1_/*c*	Mo *K*α radiation, λ = 0.71073 Å
Hall symbol: -P 2ybc	Cell parameters from 4082 reflections
*a* = 15.9660 (7) Å	θ = 4.5–26.2°
*b* = 6.8902 (3) Å	µ = 0.23 mm^−^^1^
*c* = 12.1520 (6) Å	*T* = 173 K
β = 103.381 (5)°	Block, yellow
*V* = 1300.54 (10) Å^3^	0.35 × 0.25 × 0.17 mm
*Z* = 4	

### Data collection

**Table:**

Oxford Diffraction Xcalibur diffractometer	2637 independent reflections
Radiation source: fine-focus sealed tube	2023 reflections with *I* > 2σ(*I*)
Graphite monochromator	*R*_int_ = 0.029
Detector resolution: 15.9809 pixels mm^-1^	θ_max_ = 26.3°, θ_min_ = 4.5°
ω scans	*h* = −19→18
Absorption correction: multi-scan (*CrysAlis PRO*; Oxford Diffraction, 2009)	*k* = −7→8
*T*_min_ = 0.953, *T*_max_ = 1.000	*l* = −13→15
9041 measured reflections	

### Refinement

**Table:**

Refinement on *F*^2^	Primary atom site location: structure-invariant direct methods
Least-squares matrix: full	Secondary atom site location: difference Fourier map
*R*[*F*^2^ > 2σ(*F*^2^)] = 0.036	Hydrogen site location: inferred from neighbouring sites
*wR*(*F*^2^) = 0.104	H-atom parameters constrained
*S* = 1.08	*w* = 1/[σ^2^(*F*_o_^2^) + (0.0601*P*)^2^] where *P* = (*F*_o_^2^ + 2*F*_c_^2^)/3
2637 reflections	(Δ/σ)_max_ < 0.001
158 parameters	Δρ_max_ = 0.29 e Å^−^^3^
0 restraints	Δρ_min_ = −0.19 e Å^−^^3^

### Special details

**Table:**

Geometry. All e.s.d.'s (except the e.s.d. in the dihedral angle between two l.s. planes) are estimated using the full covariance matrix. The cell e.s.d.'s are taken into account individually in the estimation of e.s.d.'s in distances, angles and torsion angles; correlations between e.s.d.'s in cell parameters are only used when they are defined by crystal symmetry. An approximate (isotropic) treatment of cell e.s.d.'s is used for estimating e.s.d.'s involving l.s. planes.
Refinement. Refinement of *F*^2^ against ALL reflections. The weighted *R*-factor *wR* and goodness of fit *S* are based on *F*^2^, conventional *R*-factors *R* are based on *F*, with *F* set to zero for negative *F*^2^. The threshold expression of *F*^2^ > 2*σ*(*F*^2^) is used only for calculating *R*-factors(gt) *etc*. and is not relevant to the choice of reflections for refinement. *R*-factors based on *F*^2^ are statistically about twice as large as those based on *F*, and *R*-factors based on all data will be even larger.

### Fractional atomic coordinates and isotropic or equivalent isotropic displacement parameters (Å^2^)

**Table:**

	*x*	*y*	*z*	*U*_iso_*/*U*_eq_	
S1	0.39851 (3)	0.51962 (7)	0.17439 (4)	0.03514 (16)	
O1	0.33687 (7)	0.25112 (16)	0.31574 (8)	0.0298 (3)	
N1	0.23682 (8)	0.61091 (19)	0.10461 (10)	0.0248 (3)	
C1	0.36863 (11)	0.7059 (3)	0.07369 (13)	0.0314 (4)	
C2	0.28401 (11)	0.7353 (2)	0.04813 (12)	0.0282 (4)	
C3	0.28957 (10)	0.4751 (2)	0.17383 (12)	0.0229 (3)	
C4	0.26767 (9)	0.3291 (2)	0.23561 (12)	0.0227 (3)	
C5	0.18289 (9)	0.2433 (2)	0.22982 (12)	0.0213 (3)	
C6	0.11844 (10)	0.2412 (2)	0.12977 (12)	0.0246 (3)	
H6	0.1286	0.3031	0.0642	0.030*	
C7	0.04027 (10)	0.1508 (2)	0.12457 (13)	0.0271 (4)	
H7	−0.0027	0.1525	0.0559	0.033*	
C8	0.02412 (10)	0.0577 (2)	0.21848 (14)	0.0297 (4)	
H8	−0.0297	−0.0040	0.2149	0.036*	
C9	0.08769 (11)	0.0561 (2)	0.31769 (13)	0.0294 (4)	
H9	0.0775	−0.0084	0.3824	0.035*	
C10	0.16563 (10)	0.1466 (2)	0.32384 (12)	0.0252 (3)	
H10	0.2083	0.1435	0.3928	0.030*	
C11	0.43748 (12)	0.8120 (3)	0.03234 (16)	0.0437 (5)	
H11A	0.4110	0.9126	−0.0216	0.065*	
H11B	0.4689	0.7204	−0.0049	0.065*	
H11C	0.4775	0.8725	0.0966	0.065*	
C12	0.23515 (12)	0.8848 (3)	−0.03065 (15)	0.0425 (5)	
H12A	0.2160	0.9881	0.0133	0.064*	
H12B	0.1849	0.8242	−0.0806	0.064*	
H12C	0.2725	0.9401	−0.0762	0.064*	
C13	0.15990 (10)	0.6830 (2)	0.13668 (13)	0.0288 (4)	
H13A	0.1086	0.6296	0.0853	0.043*	
H13B	0.1585	0.8250	0.1319	0.043*	
H13C	0.1610	0.6429	0.2144	0.043*	
C14	0.35969 (12)	0.0583 (3)	0.28898 (16)	0.0424 (5)	
H14A	0.3103	−0.0282	0.2843	0.064*	
H14B	0.4082	0.0121	0.3482	0.064*	
H14C	0.3762	0.0594	0.2162	0.064*	

### Atomic displacement parameters (Å^2^)

**Table:**

	*U*^11^	*U*^22^	*U*^33^	*U*^12^	*U*^13^	*U*^23^
S1	0.0248 (2)	0.0356 (3)	0.0462 (3)	−0.00279 (18)	0.01062 (19)	0.00815 (19)
O1	0.0287 (6)	0.0251 (7)	0.0319 (6)	0.0017 (5)	−0.0005 (5)	0.0051 (5)
N1	0.0293 (7)	0.0198 (7)	0.0265 (7)	0.0006 (6)	0.0094 (5)	0.0047 (5)
C1	0.0376 (10)	0.0279 (9)	0.0311 (9)	−0.0085 (8)	0.0129 (7)	−0.0003 (7)
C2	0.0385 (10)	0.0235 (9)	0.0241 (8)	−0.0066 (7)	0.0105 (7)	0.0006 (6)
C3	0.0223 (8)	0.0220 (8)	0.0247 (8)	0.0002 (6)	0.0061 (6)	−0.0015 (6)
C4	0.0235 (8)	0.0203 (8)	0.0237 (8)	0.0030 (6)	0.0040 (6)	0.0014 (6)
C5	0.0249 (8)	0.0150 (8)	0.0247 (8)	0.0017 (6)	0.0074 (6)	−0.0015 (6)
C6	0.0311 (9)	0.0179 (8)	0.0261 (8)	0.0042 (7)	0.0091 (6)	0.0005 (6)
C7	0.0268 (8)	0.0195 (8)	0.0326 (9)	0.0032 (7)	0.0019 (7)	−0.0051 (6)
C8	0.0269 (9)	0.0205 (9)	0.0437 (10)	−0.0023 (7)	0.0119 (7)	−0.0036 (7)
C9	0.0379 (10)	0.0217 (9)	0.0324 (9)	−0.0027 (7)	0.0161 (7)	0.0013 (7)
C10	0.0311 (9)	0.0211 (8)	0.0237 (8)	−0.0003 (7)	0.0070 (6)	0.0000 (6)
C11	0.0455 (11)	0.0442 (12)	0.0455 (10)	−0.0170 (9)	0.0192 (9)	0.0017 (9)
C12	0.0496 (11)	0.0405 (12)	0.0375 (10)	−0.0028 (9)	0.0103 (8)	0.0157 (8)
C13	0.0321 (9)	0.0225 (9)	0.0330 (9)	0.0036 (7)	0.0101 (7)	0.0026 (7)
C14	0.0347 (10)	0.0302 (11)	0.0598 (12)	0.0092 (8)	0.0059 (9)	0.0059 (9)

### Geometric parameters (Å, º)

**Table:**

S1—C1	1.7614 (17)	C8—C9	1.385 (2)
S1—C3	1.7646 (16)	C8—H8	0.9500
O1—C4	1.3999 (17)	C9—C10	1.378 (2)
O1—C14	1.435 (2)	C9—H9	0.9500
N1—C3	1.402 (2)	C10—H10	0.9500
N1—C2	1.4186 (19)	C11—H11A	0.9800
N1—C13	1.4589 (19)	C11—H11B	0.9800
C1—C2	1.330 (2)	C11—H11C	0.9800
C1—C11	1.500 (2)	C12—H12A	0.9800
C2—C12	1.497 (2)	C12—H12B	0.9800
C3—C4	1.349 (2)	C12—H12C	0.9800
C4—C5	1.464 (2)	C13—H13A	0.9800
C5—C6	1.399 (2)	C13—H13B	0.9800
C5—C10	1.404 (2)	C13—H13C	0.9800
C6—C7	1.383 (2)	C14—H14A	0.9800
C6—H6	0.9500	C14—H14B	0.9800
C7—C8	1.384 (2)	C14—H14C	0.9800
C7—H7	0.9500		
			
C1—S1—C3	90.90 (8)	C10—C9—H9	119.5
C4—O1—C14	113.50 (12)	C8—C9—H9	119.5
C3—N1—C2	112.39 (13)	C9—C10—C5	121.03 (14)
C3—N1—C13	119.55 (12)	C9—C10—H10	119.5
C2—N1—C13	119.85 (13)	C5—C10—H10	119.5
C2—C1—C11	129.13 (16)	C1—C11—H11A	109.5
C2—C1—S1	111.73 (12)	C1—C11—H11B	109.5
C11—C1—S1	119.09 (13)	H11A—C11—H11B	109.5
C1—C2—N1	114.82 (15)	C1—C11—H11C	109.5
C1—C2—C12	127.22 (15)	H11A—C11—H11C	109.5
N1—C2—C12	117.94 (15)	H11B—C11—H11C	109.5
C4—C3—N1	129.40 (14)	C2—C12—H12A	109.5
C4—C3—S1	120.65 (12)	C2—C12—H12B	109.5
N1—C3—S1	109.95 (11)	H12A—C12—H12B	109.5
C3—C4—O1	114.14 (13)	C2—C12—H12C	109.5
C3—C4—C5	129.08 (13)	H12A—C12—H12C	109.5
O1—C4—C5	116.78 (12)	H12B—C12—H12C	109.5
C6—C5—C10	117.31 (14)	N1—C13—H13A	109.5
C6—C5—C4	122.12 (13)	N1—C13—H13B	109.5
C10—C5—C4	120.40 (13)	H13A—C13—H13B	109.5
C7—C6—C5	121.24 (14)	N1—C13—H13C	109.5
C7—C6—H6	119.4	H13A—C13—H13C	109.5
C5—C6—H6	119.4	H13B—C13—H13C	109.5
C6—C7—C8	120.62 (14)	O1—C14—H14A	109.5
C6—C7—H7	119.7	O1—C14—H14B	109.5
C8—C7—H7	119.7	H14A—C14—H14B	109.5
C7—C8—C9	118.86 (15)	O1—C14—H14C	109.5
C7—C8—H8	120.6	H14A—C14—H14C	109.5
C9—C8—H8	120.6	H14B—C14—H14C	109.5
C10—C9—C8	120.94 (14)		
			
C3—S1—C1—C2	3.05 (13)	S1—C3—C4—O1	13.63 (19)
C3—S1—C1—C11	−179.13 (14)	N1—C3—C4—C5	14.8 (3)
C11—C1—C2—N1	−178.57 (16)	S1—C3—C4—C5	−166.26 (12)
S1—C1—C2—N1	−1.02 (18)	C14—O1—C4—C3	−110.21 (16)
C11—C1—C2—C12	0.3 (3)	C14—O1—C4—C5	69.70 (17)
S1—C1—C2—C12	177.82 (15)	C3—C4—C5—C6	28.4 (2)
C3—N1—C2—C1	−2.3 (2)	O1—C4—C5—C6	−151.44 (14)
C13—N1—C2—C1	146.04 (15)	C3—C4—C5—C10	−156.27 (16)
C3—N1—C2—C12	178.72 (14)	O1—C4—C5—C10	23.8 (2)
C13—N1—C2—C12	−32.9 (2)	C10—C5—C6—C7	1.3 (2)
C2—N1—C3—C4	−176.49 (15)	C4—C5—C6—C7	176.69 (14)
C13—N1—C3—C4	35.0 (2)	C5—C6—C7—C8	−0.7 (2)
C2—N1—C3—S1	4.49 (15)	C6—C7—C8—C9	−0.2 (2)
C13—N1—C3—S1	−143.98 (12)	C7—C8—C9—C10	0.6 (2)
C1—S1—C3—C4	176.64 (13)	C8—C9—C10—C5	0.0 (2)
C1—S1—C3—N1	−4.25 (11)	C6—C5—C10—C9	−0.9 (2)
N1—C3—C4—O1	−165.29 (14)	C4—C5—C10—C9	−176.45 (14)

### Hydrogen-bond geometry (Å, º)

Cg1 is the centroid of the C5–C10 ring.

**Table:**

*D*—H···*A*	*D*—H	H···*A*	*D*···*A*	*D*—H···*A*
C8—H8···*Cg*1^i^	0.95	2.83	3.6324 (16)	142
C13—H13*B*···*Cg*1^ii^	0.98	2.74	3.5657 (15)	143

Symmetry codes: (i) −*x*, *y*−1/2, −*z*+1/2; (ii) *x*, *y*+1, *z*.
